# A novel method to produce massive seedlings *via* symbiotic seed germination in orchids

**DOI:** 10.3389/fpls.2023.1114105

**Published:** 2023-03-09

**Authors:** Hua Yang, Neng-Qi Li, Jiang-Yun Gao

**Affiliations:** Institute of Biodiversity, School of Ecology and Environmental Science, Yunnan University, Kunming, Yunnan, China

**Keywords:** dust seeds, propagules, orchid conservation, seed-fungus complex, seedling production 11 4 / 33

## Abstract

Orchids produce large numbers of dust-like seeds that rely heavily on orchid mycorrhizal fungi (OMFs) for germination. Using OMFs to facilitate orchid proliferation is considered an effective method for orchid conservation but still presents challenges in practice. In this study, orchid seed-fungus complexes, in which orchid seeds and fungal mycelia were embedded together to form granules, were developed as platforms to facilitate seed germination and seedling production. Overall, seedlings were produced by seed-fungus complexes for five orchid species with large variations in the percentages of seedlings produced among species/treatments. For the different fungal treatments in *Dendrobium officinale*, Sebacinales LQ performed much better than the other fungal strains. At 90 days after sowing, 75.8±2.6% seedlings were produced in the LQ treatment, which was significantly higher than in the *Tulasnella* sp. JM (22.0±3.0%) and *Tulasnella* sp. TPYD-2 (5.3±1.0%) treatments, as well as in the LQ and TPYD-2 cocultured treatment (40.4±3.2%), while no seedlings were formed in the *Tulasnella* sp. SSCDO-5 or control treatments. For the other four orchid species, only one compatible fungus for each species was used, and the percentages of seedlings in epiphytic *Dendrobium devonianum* (67.2±2.9%) and *D. nobile* (38.9±2.8%) were much higher than those in terrestrial *Paphiopedilum spicerianum* (2.9±1.1%) and *Arundina graminifolia* (6.7±2.1%) at 90 days after sowing. Adding 1% polymer water-absorbent resin to the seed-fungus complexes of *D. officinale* seeds with fungal strain Sebacinales LQ significantly increased seedling formation, while other additional substances showed negative effects on seedling formation. For the storage of seed-fungus complexes, it is recommended to store the seed-fungus complexes in valve bags at room temperature for a short time and at a low temperature of 4°C for no more than 30 days. As a platform for symbiotic seed germination, the seed-fungus complex can facilitate seed germination, produce seedlings and support subsequent seedling growth, and its seedling productivity depends on seed germination characteristics, seed viability, and the efficiency of fungi. Seed-fungus complexes have great potential to be used as propagules in orchid conservation.

## Introduction

Orchidaceae is one of the most species-rich families of flowering plants with more than 30,000 accepted species worldwide (WCSP, 2022). Orchids are also among the most threatened of all flowering plants due to habitat loss and overcollection (Fay, 2018; Wraith and Pickering, 2019). Because they are considered flagship species for plant conservation globally (Baillie et al., 2004), orchids have received much research attention concerning all aspects of conservation from theory to practice (Fay, 2018; Phillips et al., 2020). For some rare and endangered orchid species, augmenting existing populations or establishing new populations by reintroduction/translocation is urgently needed (Swarts and Dixon, 2009; Reiter et al., 2016; Yang et al., 2020). However, the reintroduction of orchids is more challenging than that of other plants because of their strong ecological dependencies on one or more biotic interactions, requirement of mycorrhizal fungi for germination and use of specific animal pollinators (Swarts and Dixon, 2009; Fay, 2018; Liu et al., 2020).

The success of plant reintroductions can be affected by different factors, among which the type, source and size of propagules have strong impacts on the establishment of reintroduced populations (IUCN, 1998; Albrecht and Maschinski, 2012; Guerrant, 2012; Liu et al., 2015). Orchids can produce large numbers of seeds per fruit; however, their tiny dust-like seeds rely heavily on being colonized by specific mycorrhizal fungi, which provide them with the mineral and carbon resources needed to germinate (Arditti and Ghani, 2000; Dearnaley et al., 2016). This dependence on mycorrhizal fungi is also one of the major challenges for orchid conservation. Although asymbiotic germination (*in vitro* seed germination without fungal symbionts) has been commonly used to produce seedlings in many orchids for commercial production (Chen et al., 2015), symbiotic seed germination may provide a low-cost method to produce seedlings and easily maintain high genetic diversity. More importantly, for orchid reintroduction, the presence of symbiotic fungi in seedlings could result in better adaptation to the environment, leading to more successful seedling establishment and faster growth (e.g., Bustam et al., 2014; Reiter et al., 2016; Phillips et al., 2020; Yang et al., 2020; Wang et al., 2021).

Obtaining compatible fungi and using fungi to facilitate seed germination in practice are two key steps for orchid reintroduction based on symbiotic seed germination (Luo et al., 2003; Zhou and Gao, 2011). Recently, an increasing number of studies have presented *in situ/ex situ* seed baiting techniques as effective and easy ways to obtain efficient seed germination-enhancing fungi and they have been successfully used in many orchid species (e.g., Rasmussen and Whigham, 1993; Zi et al., 2014; Huang et al., 2018; Meng et al., 2019a; Meng et al., 2019b; Wang et al., 2021). However, how to use the obtained fungi to facilitate seed germination and obtain propagules for orchid reintroduction in practice are still difficult challenges. Few approaches have been reported thus far, e.g., the culturing of protocorms inoculated with mycorrhizal fungi used in *Habenaria radiata* (Takahashi et al., 2008) and the use of soil to facilitate symbiotic seed germination in *Spiranthes brevilabris* (Stewart et al., 2003). These approaches, however, are difficult to universally apply to all orchids because of the uncertainty of the fungi used, different life forms of orchids, habitat conditions and characteristics of symbiotic germination. Therefore, using mycorrhizal fungi to develop *in situ* artificial propagation techniques to facilitate reintroduction of endangered species into natural habitats is urgently needed (Luo et al., 2003; Yang et al., 2020).

In our previous study on the medicinal orchid *Dendrobium officinale* Kimura et Migo, after successfully obtaining efficient fungi for seed germination, we developed fungus-seed bags as propagules for reintroduction and restoration-friendly cultivation. Fungus-seed bags, containing mixtures of fungal powders and seeds, successfully produced seedlings with many practical advantages (Wang et al., 2021). By using this low-cost and easy-to-use method, the establishment of large amounts of seedlings from seed germination under natural conditions has been achieved successfully in *D. officinale*. It was suggested that fungus-seed bags could have universal applications for the conservation of epiphytic orchids based on symbiotic seed germination (Wang et al., 2021). However, the fungus-seed bags used as propagules for assisted colonization of terrestrial *Paphiopedilum spicerianum* (Rchb.f.) pfitzer failed to produce any seedlings under natural conditions (Yang et al., 2020). Moreover, the fungus-seed bags of *D. officinale* also failed to produce seedlings when they were released into the cracks and crevices of calcareous rocks. Fungus-seed bags were fixed on tree trunks using plastic wrap, and this simple method kept moisture inside the fungus-seed bags during seed germination, which has been considered extremely essential for seed germination and seedling formation in fungus-seed bags (Wang et al., 2021). However, this method cannot be applied in soils or on rocks for terrestrial or lithophytic orchids.

Motivated by the successes of fungus-seed bags for epiphytic orchids, we decided to develop a new method of producing seedlings *via* symbiotic seed germination, which is expected to be used as propagules for the reintroduction of terrestrial or lithophytic orchids. Inspired by artificial seeds/synthetic seeds, we proposed the concept of the orchid seed-fungus complex, in which orchid seeds and specific fungi are embedded together to form granules based on the method of creating artificial seeds (Sakamoto et al., 1992). In this study, we used seeds of three epiphytic/lithophytic orchids and two terrestrial orchids with their compatible fungi to create seed-fungus complexes and tested whether the seed-fungus complexes could be used to produce seedlings. Here, we present our results, addressing three principal questions: (1) Do seed-fungus complexes work to produce seedlings for different types of orchids? (2) What are the effects of additional substances on seedling formation of seed-fungus complexes? (3) What are the best methods and conditions for the storage of seed-fungus complexes?

## Materials and methods

### Study species and mycorrhizal fungi

A total of five orchid species were investigated in this study, including three epiphytic/lithophytic and two terrestrial orchids (**Table 1**). The three epiphytic/lithophytic orchids were *Dendrobium officinale*, *D. devonianum* Paxt. and *D. nobile* Lindl., among them, *D. officinale* and *D. nobile* mainly grow on rocks in karst landforms while *D. devonianum* grows epiphytically on trees in China. The mature fruits of three *Dendrobium* species were obtained *via* outcross-pollination trials on cultivated plants during flowering periods in 2020. For the two terrestrial orchids, *Paphiopedilum spicerianum* and *Arundina graminifolia* (D. Don) Hochr., naturally set fruits were collected from their wild populations in 2017 and 2016, respectively. For each collected fruit, seeds were carefully released and then dried and stored in the Orchid Seed Bank of Yunnan University following our previously established method (Gao et al., 2014). Prior to use, seeds were tested using the 2,3,5-triphenyl tetrazolium chloride (TTC) method (Vujanovic et al., 2000), and seed viability was recorded (**Table 1**).

**Table 1:** The seed-fungus complexes of five orchid species with eight orchid mycorrhizal fungi (OMFs) used in this study, including information on seed viability (%), the original sources of OMFs and their effects on seedling formation of host orchids reported in previous studies.

Codes of seed-fungus complexes	Orchid species and seed viability (%)	OMFs	Original sources of fungal strains	Fungal effects on seedling formation of host orchid species (Percentages of seedlings)	GenBank accession number	Deposition number	References
TP-LQ	*Dendrobium officinale* 91%	Sebacinales LQ	Protocorms and roots of *D. officinale*	70.09 ± 3.2% at 90 days after incubation	MN173026	CCTCC No. M2019744	Wang et al., 2021
TP-JM		*Tulasnella* sp. JM	Protocorms of *D. officinale*	70.29 ± 4.3% at 90 days after incubation	MN607232	OCYU-JM	Wang et al., 2021
TP-SSCDO-5		*Tulasnella* sp. SSCDO-5	Protocorms of *D. officinale*	41.10 ± 3.9% at 90 days after incubation	MH348614	OCYU-SSCDO-5	Shao et al., 2019
TP-TPYD-2		*Tulasnella* sp. TPYD-2	Seedling roots of *D. officinale*	37.12 ± 3.6% at 62 days after incubation	MN545849	CCTCC No. M2020165	Chen et al., 2022
DN-JC	*Dendrobium nobile* 35%	Sebacinales JC-1	Protocorms of *D. nobile*	34.5 ± 23.3% at 90 days after incubation	MH500251	OCYU-JC-1	Shao et al., 2020
DD-FDd1	*Dendrobium. devonianum* 95%	*Tulasnella* sp. FDd1 (*Epulorhiza* sp.)	Protocorms of *D. devonianum*	72.36 ± 11.7% at 50 days after incubation	KM226996	CGMCC No. 9551	Huang et al., 2018
PS-GYBQ01	*Paphiopedilum spicerianum* 25%	*Tulasnella* sp. GYBQ01	Protocorms of *P. spicerianum*	30.8 ± 2.6% at 90 days after incubation	MN733451	CCTCC No. M2020129	Yang et al., 2020
AG-Agp-1	*Arundina graminifolia* 50%	*Tulasnella* sp. AgP-1 (*Tulasnella* sp.)	Protocorms of *A. graminifolia*	79.75 ± 3.8% at 35 days after incubation	MK561838	GDMCC No.3672	Meng et al., 2019a

CCTCC, China Center for Type Culture Collection; OCYU, Lab of Orchid Integrative Conservation, Yunnan University, China; CGMCC, China General Microbiological Culture Collection Center; GDMCC, Guangdong Microbial Culture Collection Center.

Eight strains of orchid mycorrhizal fungi (OMFs) obtained in previous studies were used in this study to create seed-fungus complexes with seeds of corresponding orchids. The original sources, effects on seedling formation and related information about the eight strains of OMFs are also summarized in **Table 1**.

### Creating seed-fungus complexes

Orchid seeds were sterilized with 1% (w/v) sodium hypochlorite solution (NaClO) for five minutes for the three epiphytic/lithophytic orchids and 15 minutes for the two terrestrial orchids, and then washed with sterile distilled water 3-5 times. Each fungal strain used in this study was cultured in PDB liquid medium to mass produce mycelia (Yang et al., 2020; Wang et al., 2021). After mycelia were dried using sterile filter paper, 20 g mycelia and 30 g agar powder of 99% purity ((C_12_H_18_O_3_)n with Gelstrength >1700g/cm^2^) were added into 1000 ml sterile water and stirred using a homogenizer (MJ-BL35F31, Guangdong Midea Household Electrical Appliance Manufacturing Co., LTD, Foshan, China) to obtain fungal suspensions. Then, 0.13 g surface sterilized seeds, approximately 150,000 seeds amounting to 15 seeds per seed-fungus complex in the case of *Dendrobium officinale*, were added into the fungal suspensions and mixed using a magnetic stirrer for five minutes to obtain seed-fungus suspensions. To keep each seed-fungus complex containing 15 seeds for all five orchid species, 0.22 g seeds of *D. nobile*, 0.11g seeds of *D. devonianum*, 0.21g seeds of *P. spicerianum* and 0.31g seeds of *A. graminifolia* were added into 1000 ml fungal suspensions, respectively.

**Figure 1:**
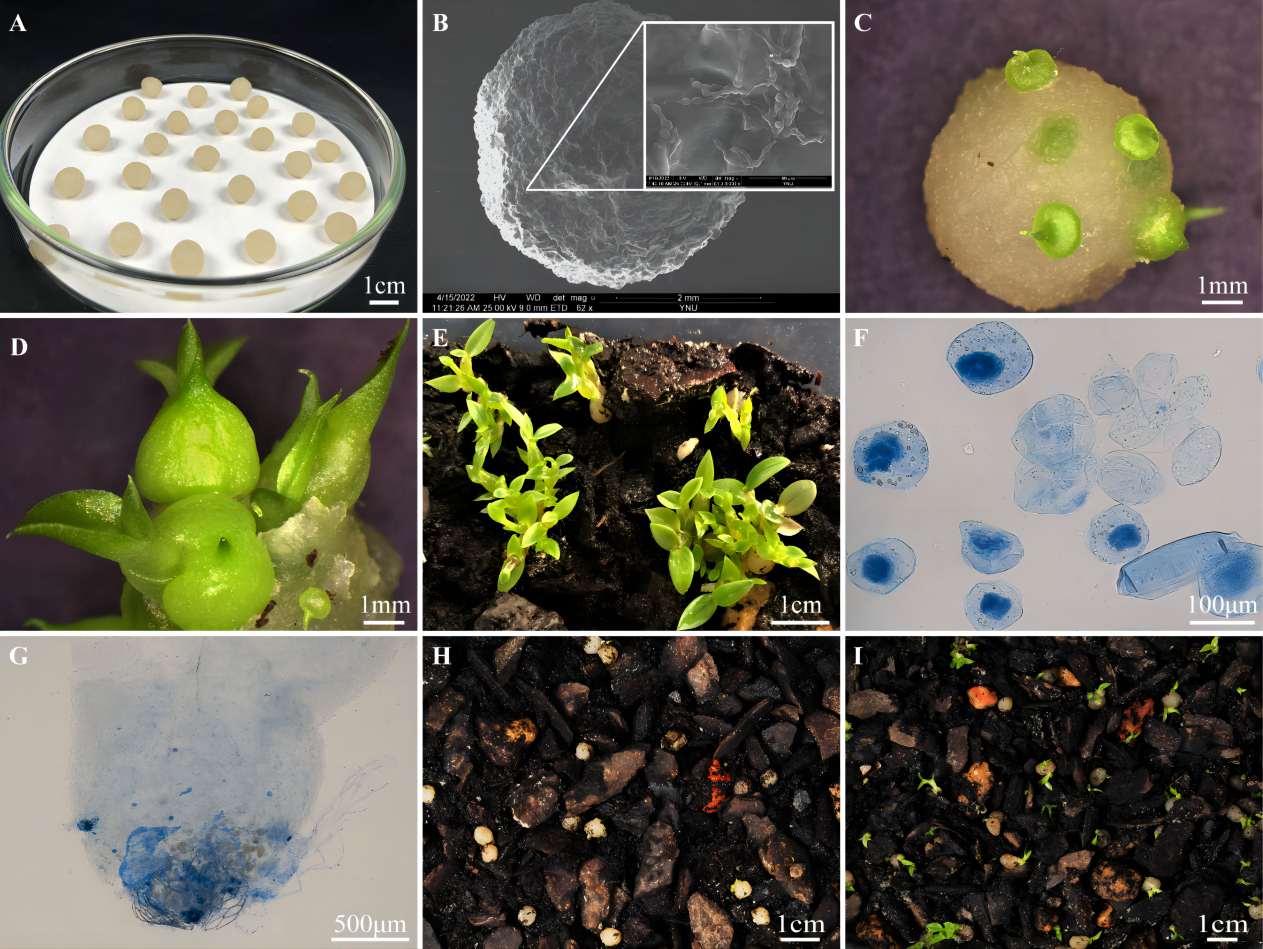
Orchid seed-fungus complexes, protocorms and seedlings produced by seed-fungus complexes at different times after sowing, and pelotons formed inside protocorms or seedling roots. **(A)** TP-LQ seed-fungus complexes containing seeds of *Dendrobium officinale* and mycelia of fungal strain LQ; **(B)** Scanning electron micrograph of a TP-LQ seed-fungus complex and amplified box showing the fungal hyphae on the surface; **(C)** Protocorms produced by a TP-LQ seed-fungus complex at 30 days after sowing; **(D)** Seedlings produced by a TP-LQ seed-fungus complex at 60 days after sowing; **(E)** A large number of seedlings with 3-5 leaves and roots produced by TP-LQ seed-fungus complexes at 90 days after sowing; **(F)** Pelotons formed inside seedling roots in TP-LQ seed-fungus complexes at 90 days after sowing; **(G)** Pelotons and fungal hyphae at the basal cells of a protocorm produced by TP-LQ seed-fungus complexes at 90 days after sowing; **(H)** Protocorms produced by TP-SSCDO-5 seed-fungus complexes at 90 days after sowing; **(I)** Seedlings produced by TP-JM seed-fungus complexes at 90 days after sowing.

In this study, seed-fungus complexes were made following the methods of artificial seeds/synthetic seeds with some modifications (Guo et al., 1996). First, the seed-fungus suspensions were added to a 3% sodium alginate solution in a 1:1 (v/v) ratio and fully shaken and mixed for 8 minutes to obtain seed-fungus embedded bodies. Then, using a 10 ml disposable syringe, the seed-fungus embedded bodies were dripped into 2% calcium chloride (CaCl_2_) solutions drop by drop for the ion exchange reaction. After solidifying for 15 minutes, seed-fungus complexes were obtained. Finally, seed-fungus complexes were transferred into sterile water for 20 minutes and removed and dried using absorbent papers. The seed-fungus complexes were spherical and relatively uniform at approximately 10 mm in diameter (**Figure 1A**).

### Efficiency of seed-fungus complexes on seedling production

For *Dendrobium officinale*, four compatible strains of fungi (LQ, JM, SSCDO-5 and TPYD-2, **Table 1**) and a mixture of LQ and TPYD-2 (mixed fungal mycelia in 1:1 g/g) were used to make the seed-fungus complexes. Additionally, seed-fungus complexes without any fungi were used as a control treatment to compare fungal effects on the seedling productivity of seed-fungus complexes. Each seed-fungus complex contained an average of 15 *D. officinale* seeds. A total of six treatments were conducted, including five fungal treatments and a control treatment. They were (1) TP-CK: control treatment without fungus; (2) TP-LQ: seed-fungus complexes of *D. officinale* with fungal strain LQ; (3) TP-JM: seed-fungus complexes of *D. officinale* with fungal strain JM; (4) TP-SSCDO-5: seed-fungus complexes of *D. officinale* with fungal strain SSCDO-5; (5) TP-TPYD-2: seed-fungus complexes of *D. officinale* with fungal strain TPYD-2; (6) TP-(LQ+TPYD-2): seed-fungus complexes of *D. officinale* with fungal strains LQ and TPYD-2. To observe the growth of fungal hyphae within the seed-fungus complexes at the initial stage, the seed-fungus complexes from the LQ treatment were freeze-dried and examined using a scanning electron microscope (SEM, FEI Quanta 650 FEG).

To assess the universal application of seed-fungus complexes, seeds of 4 other orchid species and their compatible fungi were also used to make seed-fungus complexes. The seed-fungus complexes were (1) DN-JC: *Dendrobium nobile* and fungal strain JC-1; (2) DD-FDd1: *D. devonianum* and fungal strain FDd1; (3) PS-GYBQ01: *Paphiopedilum spicerianum* and fungal strain GYBQ01; and (4) AG-AgP-1: *Arundina graminifolia* and fungal strain AgP-1 (**Table 1**).

The sterilized mixed substrates (bark, peat and volcanic stone mixed in a ratio of 2:1:1) were distributed to new plastic boxes (20.5 cm ×12.5 cm × 6.0 cm) to a depth of 3.5 cm and watered to saturation with sterile water. For each box, 30 seed-fungus complexes were sown on the surfaces of the mixed substrate and then covered. Each treatment was replicated in 12 boxes, and all boxes were incubated in a growth chamber (RXZ300B, Ningbo Southeast Instruments Co., Ltd, Ningbo, China) at 25±2 °C and a 12 h/12 h light/dark cycle. Seed germination status for each treatment was monitored and recorded regularly. At 30, 60 and 90 days after sowing, seedlings in each box of all treatments were counted. Protocorms, seedlings and roots of different treatments were randomly sampled and stained following the methods described by Phillips and Hayman (1970) to observe their fungal colonization statuses under a microscope (DM2000, Leica Microsystems GmbH, Wetzlar, Germany).

### Effects of additional substances on seedling formation

According to the results of the above experiments, the best performing seed-fungus complex for *D. officinale* was that of the TP-QL treatment and it was used to evaluate the effects of additional substances on seedling formation. A total of 7 substances that are commonly used in artificial seeds were added to the TP-LQ seed-fungus complex. The treatments were as follows: (1) LQ-CK: control treatment without substance added; (2) LQ-S: 1% sucrose added; (3): LQ-PWAR: 1% polymer water-absorbent resin (sodium polyacrylate, (C3H3NaO2)n, 0.50-0.80g/cm^3^ of density, produced by Shandong Youso Chemical Technology Co., LTD, Linyi, China) added; (4) LQ-TS: 1.5% tapioca starch added; (5) LQ-T: 1% trehalose added; (6) LQ-C: 1% chitosan added; (7) LQ-AC: 0.1% activated carbon added; and (8) LQ-SB: 0.2% sodium benzoate added. Sowing and culture conditions of the seed-fungus complexes were the same as those used for the previous eight treatment experiment, and each treatment was also replicated in 12 boxes. At 50, 70 and 90 days after sowing, seedlings were counted in each box of all treatments.

### Storage of seed-fungus complex

To assess the effects of storage methods and conditions on seedling formation, TP-LQ seed-fungus complexes were stored in valve bags (VB), sealed tubes (ST) and valve bags after freeze-drying (VB-FD) at 4°C and 25°C for 0, 15, 30 and 45 days. A total of 24 treatments (3 methods * 2 temperatures * 4 storage times) were conducted. For each treatment, five replications with 60 seed-fungus complexes each were carried out. At each time point after storage, seed-fungus complexes from different treatments were sown and cultured using the same methods and under the same conditions as the previous experiments. At 60 days after sowing, seedlings were counted in each box of all 24 treatments.

### Data collection and statistical analysis

In this study, seedling was defined as the emergence of the first leaf and further development. The percentage of seedlings (S) was calculated as S = s/t, s = number of seedlings, and t = average number of seeds per seed-fungus complex. A nonparametric one-sample K-S test was used to determine whether the data in each group obeyed a normal distribution. One-way ANOVAs and least significant difference (*LSD*) tests were used in cases when data conformed to a normal distribution and generalized linear models (*GLMs*) when data did not meet a normal distribution to determine the effects of different treatments on seedling development at different time points. All statistical analyses were performed in SPSS (version 26.0). The results are expressed as the mean ± standard error (mean ± SE), and the alpha-type I error was fixed at 5% (thus, all nonsignificant differences have *P* > 0.05).

## Results

### Efficiency of seed-fungus complexes on seedling production

For *Dendrobium officinale*, the SEM study clearly showed that fungal hyphae of the LQ strain could grow inside the seed-fungus complex (**Figure 1B**). After sowing, seedlings were produced in the four fungal treatments of TP-LQ, TP-JM, TP-TPYD-2 and TP-(LQ+TPYD-2) with great variations in the percentage of seedlings at different time points among treatments, while no seedlings occurred in the TP-SSCDO-5 fungal treatment or control treatment (TP-CK).

**Figure 2:**
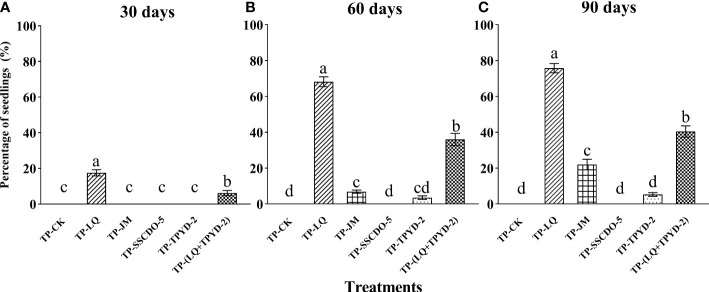
The percentages of seedlings (mean±SE) in the control (TP-CK) and five fungal treatments (LQ, JM, SSCDO-5, TPYD-2, and LQ+TPYD-2) of seedfungus complexes of *Dendrobium officinale* at **(A)** 30, **(B)** 60, and **(C)** 90 days after sowing. In each panel, different letters indicate significant differences (*p* < 0.05) based on one-way ANOVA and the least significant difference (*LSD*) method where the data are normally distributed and the generalized linear model (*GLM*) where the data are not normally distributed.

At 30 days after sowing, many protocorms could be clearly observed within seed-fungus complexes of all treatments except for the TP-SSCDO-5 and TP-CK treatments (**Figure 1C**). Seedlings were found only in the TP-LQ and TP-(LQ+TPYD-2) treatments, and the percentage of seedlings in the TP-LQ treatment (17.6±1.7%) was significantly higher than that in the TP-(LQ+TPYD-2) treatment (6.2 ± 1.4%; *P* < 0.01; **Figure 2A**).

At 60 days after sowing, *D. officinale* seedlings grew from seed-fungus complexes in all treatments except for the TP-SSCDO-5 and TP-CK treatments, in which no protocorms or seedlings were found. The percentages of seedlings were significantly different among treatments (F = 222.52, *P* < 0.001; **Figure 2B**). The percentage of seedlings in the TP-LQ treatment (68.2±2.7%) was significantly higher than that in any other treatment, followed by the TP-(LQ+TPYD-2) treatment (36.0±3.4%), TP-JM treatment (6.9±0.9%) and TP-TPYD-2 treatment (3.6±1.0%). There was no significant difference between the TP-JM and TP-TPYD-2 treatments (*P* = 0.21; **Figure 2B**). At this stage, most seedlings in the TP-LQ treatment bore second leaves (**Figure 1D**).

At 90 days after sowing, most seedlings in the TP-LQ treatment developed 3-5 leaves with strong roots (**Figure 1E**), and pelotons could be clearly observed inside the root cells (**Figure 1F**). Fungal hyphae congregated at the basal end outside of the protocorms that did not develop into seedlings (**Figure 1G**). At this stage, protocorms were formed in the TP-SSCDO-5 treatment (**Figure 1H**), and seedlings in the TP-JM treatment developed 2-3 leaves (**Figure 1I**), but *D. officinale* seeds did not germinate in the TP-CK treatment. The percentages of seedlings significantly differed among the different treatments (F = 202.88, *P* < 0.001; **Figure 2C**), in which 75.8±2.6% seedlings in the TP-LQ treatment was still significantly higher than the percentages of the other treatments, while 40.4 ±3.2% seedlings in the TP-(LQ+TPYD-2) treatment was significantly higher than the percentages of the TP-JM treatment (22.0±3.0%) and the TP-TPYD-2 treatment (5.3 ± 1.0%) (all *P* < 0.001; **Figure 2C**).

For the other four orchid species, after sowing, seedlings were produced in seed-fungus complexes of all treatments with various effectiveness. Finally, at 90 days after sowing, the percentages of seedlings were much higher for the two epiphytic orchids (*Dendrobium devonianum*: 67.2±2.9%; *D. nobile*: 38.9±2.8%) than the two terrestrial orchids (*Paphiopedilum spicerianum*: 2.9±1.1%; *Arundina graminifolia*: 6.7±2.1%; **Table 2**).

**Table 2:** Seedling formation at 30, 60 and 90 days after sowing of seed-fungus complexes in four orchid species.

Seed-fungus complexes	Percentages of seedlings (mean ± SE%)		
	30 days after sowing	60 days after sowing	90 days after sowing
DN-JC	17.6±1.8%	29.6±2.2%	38.9±2.8%
DD-FDd1	5.6±1.0%	63.0±2.4%	67.2±2.9%
PS-GYBQ01	0.0±0.0%	1.9±0.9%	2.9±1.1%
AG-AgP-1	6.3±1.6%	3.8±1.7%	6.7±2.1%

DN-JC, seeds of *Dendrobium nobile* with fungal strain JC-1; DD-FDd1, seeds of *D. devonianum* with fungal strain FDd1; PS-GYBQ01, seeds of *Paphiopedilum spicerianum* with fungal strain GYBQ01; AG-AgP-1, seeds of *Arundina graminifolia* with fungal strain AgP-1.

### Effects of additional substances on seedling formation

**Figure 3:**
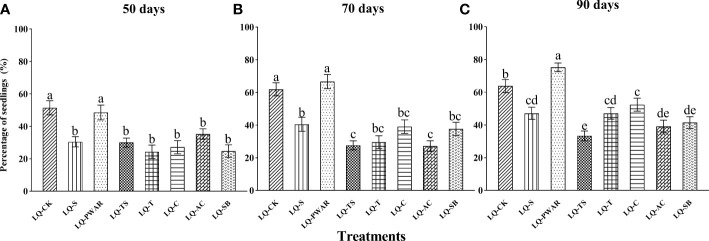
Effects of additional substances on seedling formation of seed-fungus complexes of *Dendrobium officinale* with fungal strain LQ at **(A)** 50, **(B)** 70 and **(C)** 90 days after sowing. LQ-CK, seed-fungus complexes of *D. officinale* with fungal strain LQ; LQ-S, 1% sucrose added; LQ-PWAR, 1% polymer water-absorbent resin added; LQ-TS, 1.5% tapioca starch added; LQ-T, 1% trehalose added; LQ-C, 1% chitosan added; LQ-AC, 0.1% activated carbon added; LQ-SB, 0.2% sodium benzoate added. The significant differences in the percentages of seedlings (mean±SE) among different treatments at 50, 70 and 90 days after sowing are shown by different lowercase letters at *p* < 0.05.

Compared to the control treatment (LQ-CK), only the seed-fungus complexes with 1% polymer water-absorbent resin (LQ-PWAR) added produced a significant number of seedlings, while the other six substances added to seed-fungus complexes significantly reduced seedling formation (**Figure 3**). At 50 days after sowing, the percentages of seedlings were significantly different among all treatments (F = 7.55, *P* < 0.001; **Figure 3A**). The percentages of seedlings in both the LQ-CK treatment (51.4±4.4%) and LQ-PWAR treatment (48.6±4.6%) were significantly higher than those in the other six treatments (all *P* < 0.05), but there was no significant difference between the LQ-CK and LQ-PWAR treatments (*P* = 0.60) or among the six treatments (all *P* > 0.05). At 70 days after sowing, the percentages of seedlings in the LQ-PWAR treatment (66.7 ± 4.4%) were higher than those in the LQ-CK treatment (61.9 ± 4.0%), but there was no significant difference in the percentages of seedlings between the two treatments (*P* = 0.39; **Figure 3B**). The other six substances added to seed-fungus complexes all showed a significantly negative effect on seedling formation compared with the control treatment (all *P* < 0.001; **Figure 3B**). At 90 days after sowing, the LQ-PWAR treatment yielded the highest seedling formation rate (75.2 ± 2.7%), which was significantly higher than that in the control treatment (LQ-CK: 63.8 ± 4.0%; *P* < 0.05), while the percentages of seedlings in the other six substance treatments were all significantly lower than those in the control treatment (all *P* < 0.05; **Figure 3C**).

### Storage of seed-fungus complexes

**Figure 4:**
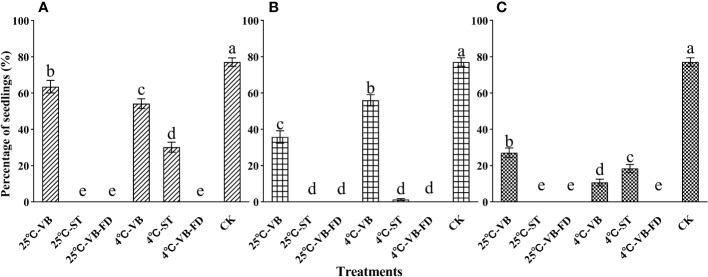
The percentages of seedlings (mean±SE) in different storage treatments (storage methods * temperatures * storage time) of seedfungus complexes of Dendrobium officinale with fungal strain LQ at 60 days after sowing. The significant differences among storage methods at 4°C and 25°C for **(A)** 15, **(B)** 30 and **(C)** 45 days are shown by different lowercase letters at p < 0.05. VB, stored in valve bags; ST, stored in sealed tubes; VB-FD, stored in valve bags after freeze-drying; CK, TP-LQ seed-fungus complexes were sown without storage.

Storage methods, storage time and storage temperatures all had significant impacts on seedling formation of seed-fungus complexes (three-way ANOVA; storage methods: F = 330.65, storage time: F = 54.33, storage temperatures: F = 16.77; all *P* < 0.001). No seedlings were produced by seed-fungus complexes in VB-FD treatment after freeze-drying. For the VB and ST treatments, seedling formation decreased with increasing storage time. At 60 days after sowing, the percentage of seedlings in the VB and ST treatments/conditions was significantly lower than that in the control treatment (**Figure 4**).

For seed-fungus complexes stored for 15 days, the percentages of seedlings in the VB treatment at 25°C (63.6±3.5%) were significantly higher than those in the same treatment stored at 4°C (54.2±2.7%) and in the ST treatment at 4°C (30.2±2.7%), but all were significantly lower than those in the control treatment (77.1±2.3%; all *P* < 0.01); no seedlings grew in the other treatments/conditions (**Figure 4A**). For a storage time of 30 days, the percentages of seedlings in the VB treatment at 4°C (56.0±3.1%) were significantly higher than those in the same treatment stored at 25°C (35.8±3.4%), and both were significantly lower than those in the control treatment (all *P* < 0.01). However, only a few seedlings grew in the ST treatment at 4°C (1.3±0.5%), and no seedlings grew in the other treatments/conditions (**Figure 4B**). At a storage time of 45 days, seedlings were only found in the above three treatments/conditions, but the percentages of seedlings decreased greatly compared with those at a storage time of 30 days. The percentages of seedlings in the VB treatment at 25°C (27.1±2.6%) were still significantly higher than those in the VB treatment stored at 4°C (10.7±1.9%) and in the ST treatment at 4°C (18.4±2.2%), and all were significantly lower than those in the control treatment (all *P* < 0.01; **Figure 4C**).

## Discussion

Orchids are characterized by producing tiny dust-like seeds with a small and undeveloped embryo not surrounded by an endosperm, which will only germinate and develop in the presence of symbiotic fungi (Arditti and Ghani, 2000; Rasmussen and Rasmussen, 2014). Although orchids produce an enormous quantity of seeds, limited studies have suggested that seedling recruitment is extremely low (less than 1%) in natural populations (Batty et al., 2001). The germination characteristics make orchid conservation a difficult task, especially for orchid reintroduction, which requires appropriate propagules (Zhou and Gao, 2011). However, because each seed acts as an independent propagule, the large number of seeds may make it possible to restore all threatened orchids if effective fungi for seed germination are obtained and an appropriate way to facilitate seed germination is found (Zhou and Gao, 2011).

### Efficiency of seed-fungus complexes on seedling production

In this study, seed-fungus complexes containing orchid seeds and corresponding fungi were developed as propagules to facilitate orchid proliferation. As expected, seedlings were produced by seed-fungus complexes for all 5 species of orchids investigated. However, the percentages of seedlings at 90 days after sowing varied greatly among different species and treatments. For the two terrestrial orchids, seedling formation was not only lower than for the three epiphytic *Dendrobium* species (**Table 2**) but also much lower than those reported for the same orchid species incubated with the same fungi on OMA medium in our previous studies (*Paphiopedilum spicerianum*: 2.9±1.1% vs. 30.8 ± 2.6%; *Arundina graminifolia*: 6.7±2.1% vs. 79.75 ± 3.8% at 35 days after incubation; **Table 1**). Two possible reasons could explain the low seedling formation of the two terrestrial orchids in this study. One is that the seed viability of the two terrestrial orchids was greatly reduced after long-term storage compared to the time points we used in previous studies (Meng et al., 2019a; Yang et al., 2020), in which seed viability of *P. spicerianum* decreased from > 90% in 2017 to 25%, while seed viability of *A. graminifolia* decreased from > 95% in 2016 to 50% (**Table 1**). However, the responses of different orchid species to dark and light conditions vary greatly during germination (Arditti, 1967; Arditti and Ernst, 1984). For epiphytic *Dendrobium* species, seeds can germinate and form protocorms in the absence of light, but seedling development requires exposure to light (Wang et al., 2011; Zi et al., 2014; Huang et al., 2018), while some studies have suggested that dark conditions can stimulate and improve germination in some terrestrial orchids (Harvais, 1973; Stewart and Kane, 2006). In a previous study, seeds of *Paphiopedilum spicerianum* were incubated with the same fungus, *Tulasnella* sp. GYBQ01 on OMA medium in the dark at 25 ± 2°C for 8 weeks followed by a 12 h/12 h light/dark cycle, and 30.8 ± 2.6% of seeds germinated and grew into seedlings at 90 days after incubation (Yang et al., 2020). However, in the current study, we failed to set a dark period for the seed-fungus complexes of *P. spicerianum* at the beginning of sowing, which may have resulted in a low seedling formation rate.

*Dendrobium officinale* is widely distributed in subtropical areas of China and Japan with diverse habitat conditions. Orchids seem to recruit OMFs from endophytes colonizing roots (Selosse et al., 2022), the variation in mycorrhizal associations in orchids is to some extent affected by specific environmental conditions (Smith and Read, 2008; Shefferson et al., 2019). Ecologically, the natural occurrences of OMFs can affect key processes such as seed germination and plant growth and survival (Jacquemyn et al., 2012) and therefore result in the compatibility of mycorrhizal associations in orchids (Põlme et al., 2018). The four OMFs used to create seed-fungus complexes in this study were originally from different geographical locations/habitats (**Table 1**) and have been shown to be compatible with *D. officinale* and quickly promote seed germination up to the seedling stage with relative effectiveness (Shao et al., 2019; Wang et al., 2021; Chen et al., 2022). Although seedlings were produced in the three fungal treatments, the seedling productivity varied greatly among treatments (**Figure 2**). Sebacinales LQ performed much better than other fungi, and its hyphae could vigorously grow inside the seed-fungus complex (**Figure 1B**). The percentage of seedlings in the TP-LQ treatment at 90 days after sowing was even higher than those incubated with the same fungi on OMA medium in previous studies (75.8±2.6% vs. 70.09 ± 3.2%) (Wang et al., 2021), while seedling formation in the other three fungal treatments was much worse than previously reported for the same orchid species incubated with the same fungi on OMA medium (**Table 1**). Because different OMFs are able to use different nutrient resources (Pellegrino et al., 2014), it has been suggested that fungal hyphal growth is influenced by nutrient media and in turn affects symbiotic seed germination (Figura et al., 2021; Mujica et al., 2021). In the current study, fungal mycelia were embedded in the seed-fungus complex. Once hyphae grew out of the seed-fungus complex, fungi could use sources of carbon and other nutrients from the mixed substrates, but hyphal growth at the initial stage could be influenced by the seed-fungus complex and therefore result in variations in seedling productivity in different fungal treatments.

The two fungal strains LQ and TPYD-2 were used together in the TP-(LQ+TPYD-2) treatment, but the percentages of seedlings at different time points after sowing were all significantly lower than those in the TP-LQ treatment (**Figure 2**). The results are consistent with those of previous studies, in which fungal cocultures have been observed to result in significantly lower seed germination, protocorm formation and seedling development than monocultures (Sharma et al., 2003; Ma et al., 2022). Such reduced efficiency as a result of functional redundancy between fungal species was also found in arbuscular mycorrhizal fungi and explained as induced competition (Thonar et al., 2014; Gosling et al., 2016; Crossay et al., 2019). The occurrence of antagonism or competition between mycorrhizal fungi has been suggested in *D. officinale* (Ma et al., 2022).

### Effects of additional substances on seedling formation

In this study, orchid seed-fungus complexes were created following the method of artificial seeds/synthetic seeds. However, a seed-fungus complex is completely different from an artificial seed. It contains a number of orchid seeds and fungal mycelia and serves as a platform for symbiotic seed germination. The preliminary function of the seed-fungus complex is to ensure the survival of seeds and fungi in natural conditions. Orchid seeds need to avoid desiccation before germination (Rasmussen et al., 2015). In our previous study, using plastic wrap to keep fungus-seed bags moist was also considered essential for seed germination and seedling formation (Wang et al., 2021). Sodium alginate, as the main material used in seed-fungus complexes, has the advantages of strong water retention and permeability, biological activity, low cost and nontoxicity and has been widely used in artificial seeds. Some studies have suggested that different substances added to artificial seeds can increase embryo viability and in turn promote embryo germination (e.g., Timbert et al., 1996; Zhang and Li, 2009).

A total of 7 substances were added to seed-fungus complexes, which were developed in the current study for the first time, to assess their effects on seedling formation in *D. officinale* with the fungal strain LQ. Only 1% polymer water-absorbent resin (LQ-PWAR treatment) significantly increased seedling formation compared with the control treatment at 90 days after sowing (**Figure 3**). The addition of polymer water-absorbent resin may improve the water retention and water absorption of seed-fungus complexes, which has a positive effect on seedling formation. This result also confirmed the importance of water retention for seed-fungus complexes during symbiotic seed germination. In the other six treatments with different added substances, the percentages of seedlings at different time points after sowing were all significantly lower than those in the control treatment (**Figure 3**). The additional substances may change the structure of seed-fungus complexes, as has been considered in artificial seeds (Huang et al., 1995). We also observed that the seedlings in some treatments were stronger than those in the control treatment, and the effects of additional substances on the qualities of seedlings need to be assessed in detail in future studies. Moreover, the effects of adding two or more substances on seedling formation and seedling quality should also be considered.

### Storage of seed-fungus complex

Orchid seed-fungus complexes are expected to be used as propagules for reintroduction. In practice, the created seed-fungus complexes may not be used within enough time, and therefore, how to properly store seed-fungus complexes is also essential for broad applications in orchid conservation. Seed-fungus complexes have a high water content and easily lose water and shrink. Moreover, as a carrier of seeds for germination, storage of the seed-fungus complex should maintain the vitality of both orchid seeds and fungi. For the storage of artificial seeds, dehydration and low temperature are commonly applied (e.g., Gray, 1989; Takahata et al., 1993; Krishna-Kumar and Thomas, 2012). In this study, we tested three storage methods for orchid seed-fungus complexes at room temperature (25°C) and low temperature (4°C). No seedlings were found in the VB-FD treatment, indicating that fungi, seeds, or both lost viability after freeze-drying. Overall, seed-fungus complexes stored in valve bags were better off than those stored in sealed tubes. For short-term storage of no more than 15 days, it is recommended to store seed-fungus complexes of *D. officinale* with fungal strain LQ in valve bags at room temperature, and for storage of no more than 30 days, seed-fungus complexes should be stored in valve bags at a low temperature of 4°C. Based on the current results, it is not recommended to store the seed-fungus complexes for longer than 30 days.

## Conclusion

The seed-fungus complexes developed in this study successfully facilitated seed germination, produced sizable amounts of seedlings and supported subsequent seedling growth in all five investigated orchid species. As a carrier of seeds and fungi for symbiotic seed germination, seedling productivity depends on seed germination characteristics, seed viability, and the efficiency of the fungi used. Adding 1% polymer water-absorbent resin to seed-fungus complexes significantly increased seedling formation rate because it likely improved the water retention and water absorption of the seed-fungus complexes. It is recommended to store the seed-fungus complexes in valve bags at room temperature for a short time and at a low temperature of 4°C for no more than 30 days. Seed-fungus complexes have great potential to be used as propagules in orchid conservation with many practical advantages, e.g., low-cost mass seedling production, convenient transportation, controllable seedling quantity and density, ease of use in the field, and environmentally friendly biodegradable materials.

## Data availability statement

The original contributions presented in the study are included in the article/supplementary material. Further inquiries can be directed to the corresponding author.

## Author contributions

J-YG and HY designed the experiment. HY and N-QL conducted experiments. HY and J-YG performed the analysis. J-YG and HY wrote the wrote and reviewed manuscript. All authors contributed to the article and approved the submitted version.
